# Standard and advanced echocardiographic study of patients with Paget's disease of bone: Evidence of a pagetic heart disease?

**DOI:** 10.1111/joim.20069

**Published:** 2025-05-08

**Authors:** Alfonso Giaquinto, Veronica Abate, Anita Vergatti, Riccardo Muscariello, Adelaide Iervolino, Martina Pucci, Guido Cavati, Filippo Pirrotta, Gianpaolo De Filippo, Roberta Esposito, Lanfranco D'Elia, Daniela Merlotti, Luigi Gennari, Domenico Rendina

**Affiliations:** ^1^ Department of Clinical Medicine and Surgery Federico II University Naples Italy; ^2^ Department of Medicine, Surgery and Neurosciences University of Siena Siena Italy; ^3^ Assistance Publique‐Hôpitaux de Paris, Hôpital Robert‐Debré Service d'Endocrinologie‐Diabétologie Paris France

**Keywords:** echocardiography, heart failure, Paget's diseases of bone, pagetic heart disease, speckle tracking

## Abstract

**Background:**

Paget's disease of the bone (PDB) is a metabolic bone disorder involving one or more skeletal sites. Cardiovascular diseases (CVDs) have been described in patients with PDB but have not been systematically analysed.

**Objectives:**

This study aimed to compare standard and advanced (speckle‐tracking) echocardiographic parameters measured in patients with PDB and controls matched for age, weight, height and history of hypertension but without metabolic bone disorders.

**Methods:**

This multicentre case–control study included all patients with PDB referred to the Federico II and Siena Universities, Italy, from March 2019 to October 2022. During the same time, we enrolled at least one control for each patient, matched for age, sex, body mass index (BMI) and history of hypertension.

**Results:**

Sixty‐nine patients with PDB and 115 healthy controls were enrolled in this study. All patients with PDB were treated with zoledronic acid at the time of diagnosis. Compared with controls, on standard echocardiography, patients with PDB showed a high prevalence of aortic and mitral valve calcifications and/or sclerosis, reduced left ventricular (LV) ejection fraction, stroke volume, cardiac output, increased interventricular septum thickness, posterior wall thickness, LV mass index, relative wall thickness, relative diastolic wall thickness, *E*/*e*′ ratio and systemic vascular resistance. Using speckle‐tracking echocardiography, patients with PDB showed a lower global longitudinal strain and global myocardial work efficiency than controls. There was no relationship between the PDB activity and extent and severity of cardiac abnormalities.

**Conclusion:**

Overall, the myocardial function and structure were impaired in patients with PDB. Additionally, PDB was associated with early subclinical myocardial damage.

## Graphical abstract

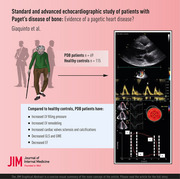

## Introduction

Paget's disease of the bone (PDB, OMIM 602080) is the most common metabolic bone disorder worldwide after osteoporosis [1]; Large variations have been reported in terms of its prevalence in ethnic and geographical distributions [2, 3, 4]. PDB is a chronic and focal metabolic bone disorder characterized by increased and disorganized bone turnover affecting one or more skeletal sites, that is, monostotic and polyostotic PDB, respectively. PDB affects both sexes, with a slight predominance in men, and is primarily observed in middle‐aged or older adults [5, 6]. PDB pathogenesis is related to interactions between environmental and genetic factors. Among the latter, *p62/sequestosome 1* (*SQSTM1*) mutations have been described in approximately 25%–40 % of familial cases and 10%–15% of sporadic cases [7]. More recent research supports the hypothesis that PDB is a multisystemic and polygenic disease [8, 9]. A study conducted on mice reported that *p62* mutation plays a crucial role in the activation of autophagy in cardiomyocytes, possibly impairing cardiac function [10].

According to the World Health Organization, cardiovascular disease (CVD), that is, coronary artery disease (CAD), cerebrovascular disease and peripheral artery disease, are the leading causes of death worldwide [11].

Globally, CVD and metabolic bone disorders, such as PDB, are mainly observed in adult and elderly populations and represent two of the major public health concerns. In addition, their social and economic burdens have steadily increased owing to the ageing world population [12, 13]. In addition to epidemiological data, in the last few decades, clinical and experimental studies have indicated a common etiopathogenic pattern between metabolic bone disorders and CVD, providing evidence of a bone‐cardiovascular axis [14, 15, 16]. In this regard, the relationship between SQSTM‐1 intracellular functions and CVD pathogenesis has been demonstrated in both in vitro and in vivo models [10, 17].

Therefore, it is obvious that some authors consider CVDs as a common non‐neoplastic complication in patients with PDB [5, 6, 18]. However, heart pathologies have only been described anecdotally and have not been systematically analysed in patients with PDB [19, 20]. In particular, only a few studies have analysed cardiac complications in patients with PDB using standard echocardiographic examinations, and only two of them had a case–control design [21, 22]. In recent times, new techniques and prognostic parameters, such as the speckle‐tracking echocardiography (STE), have become available [23]. STE is an ultrasound imaging technique based on the analysis of the spatial dislocation (tracking) of speckles. The latter are generated by the interaction between the ultrasound beam and myocardial fibres in routine bi‐dimensional sonograms [24]. STE guarantees objective and quantitative evaluation of global and regional myocardial function by analysing myocardial deformation in three spatial directions (longitudinal, radial and circumferential). In addition, STE evaluates the occurrence, direction and velocity of left ventricle (LV) rotation [25]. STE allows an in‐depth evaluation of myocardial systolic and diastolic functions in a broad range of pathological conditions, such as arterial hypertension, diabetes mellitus, CAD, valvular heart disease, heart failure and cardiomyopathies, ensuring greater sensitivity and specificity than traditional echocardiographic techniques and comparable to those of magnetic resonance imaging [24].

We conducted this case–control study to evaluate the conventional and speckle‐tracking echocardiographic parameters in patients with PDB and controls matched for age, sex, body mass index (BMI) and history of arterial hypertension. In patients with PDB, we also evaluated the relationship between echocardiographic parameters (both conventional and speckle tracking), PDB extension and metabolic activity using bone scintigraphy and total alkaline phosphatase serum levels, respectively. Additionally, in patients with PDB, we eventually evaluated the potential influence of *SQSTM1* mutations on conventional and STE parameters.

## Patient and methods

All patients with PDB referred to the Department of Clinical Medicine and Surgery of Federico II University in Naples and the Department of Medicine, Surgery, and Neurosciences of Siena University in Siena from March 2019 to October 2022 were considered eligible for this study. Both departments are the main national centres for PDB management and have shared diagnostic and therapeutic protocols for several years. PDB diagnosis was based on radiological and scintigraphic evidence in all cases [5]. Based on bone scintigraphy [17], patients with PDB were divided into monostotic (a single‐skeletal site involved) and polyostotic (more than one skeletal site involved) groups. Simultaneously, we enrolled control patients matched with patients with PDB in terms of age, sex, BMI and history of arterial hypertension. During the same period that is from March 2019 to October 2022, controls were enrolled among patients referred to the Excellence Center for Hypertension and Interdepartmental Laboratory of Echocardiography of Federico II University, employers and interns of Federico II University and in‐laws and non‐genetically related relatives of patients with PDB.

### Exclusion criteria

To rule out any interference from other causes of cardiac impairment in both patients with PDB and controls, individuals in both groups meeting the following criteria were excluded: heart rate ≥ 100 beats per minute (bpm) [26]; history of CAD; acute myocardial infarction; stroke; transient ischaemic events [27]; stages B–D of valvular heart diseases according to American College of Cardiology/American Heart Association criteria [28]; stages B–D of heart failure according to American College of Cardiology/American Heart Association criteria [29]; primary cardiomyopathies [30]; congenital heart diseases [31]; ventricular and atrial arrhythmias [32]; diabetes mellitus [33]; dyslipidaemia [34]; estimated glomerular filtration rate (eGFR) calculated using the EPI‐CKD formula [35] <60 mL/min/1.73 m^2^ [36]; active or previous history of cancer and poor‐quality echocardiograms. Additional exclusion criteria for controls were personal or family history of PDB, rickets [37], evidence of bone deformity and short stature (individual's height ≤ 3rd percentile for the mean height of a given age, sex and population group) [38].

### Biochemical and genetic parameters

In patients with PDB, in addition to anthropometric parameters, we measured the serum total alkaline phosphatase (tALP) levels to identify the metabolic activity of the disease. The PDB is considered metabolically active when patients present with serum tALP levels above the laboratory reference range [39]. Serum tALP levels are expressed as percentages relative to the upper limits of the laboratory reference range. *SQSTM1* analysis was performed as previously described [40].

### Echocardiographic procedures

Doppler echocardiographic examinations were performed using Vivid E95 ultrasound machines (GE Vingmed Ultrasound, Horten, Norway) equipped with a 2.5‐MHz transducer with harmonic capability, and the echocardiography report was prepared according to the European Association of Cardiovascular Imaging (EACVI) standardization [41]. Quantitative analyses were performed according to published guidelines [42]. Before the examination, a physician blinded to the diagnosis measured the cuff brachial blood pressure (BP) (the mean of three measurements) and heart rate from an electrocardiogram trace obtained during apical long‐axis view recording. Left ventricular ejection fraction (LVEF) and LV volume were measured according to published guidelines, and LVEF < 53% was considered on the lower side [19]. The LV mass and left atrial volume were indexed to body surface area (BSA), and LV mass indices >95 g/m^2^ in women and >115 g/m^2^ in men were defined as the presence of LV hypertrophy [19]. Doppler‐derived transmitral inflow early (*E*) and atrial (*A*) peak velocities, *E*/*A* ratio, *E*‐velocity deceleration time, pulsed tissue Doppler of the septal and lateral annuli (early diastolic velocity [*e*′]), average *E*/*e*′ ratio and tricuspid regurgitation jet peak velocity were determined in the apical four‐chamber view according to the 2016 recommendations [43]. Pulmonary artery systolic pressure was based on the tricuspid regurgitation peak velocity with the application of the simplified Bernoulli equation and addition of the right atrial pressure. The right atrial pressure was estimated according to the American Society of Echocardiography guidelines [44]. The presence and grade of valvular heart disease were evaluated using an integrated approach according to the European recommendations [45]. STE was performed as published procedures [46, 47]. The LV longitudinal deformation was recorded on 2D images of three consecutive cardiac cycles from three apical (long‐axis, four‐ and two‐chamber) views at approximately equal heart rates. The interactive software of a dedicated workstation (Echopac BT13 version, GE Healthcare) allowed automatic tracing of the endocardial‐cavity interface with possible subsequent manual adjustment and rejection of segments with poor imaging quality. Each of the three apical images was automatically divided into six myocardial segments. Peak negative longitudinal strain was measured from six segments in each of the three apical views, and the global longitudinal strain was computed as the average of the individual peak strains before aortic valve closure. The reproducibility of the speckle‐tracking echo in our laboratory has been previously reported [48]. Global longitudinal strain was considered an absolute value according to the Chamber Quantification recommendations [42]. Quantitative analysis of myocardial function was performed using a commercially available vendor‐specific software package (Echopac V. 2.03, GE Healthcare), according to standardized procedures [23]. The cuff systolic BP was assumed to be equal to the LV peak systolic pressure and combined with the acquired global longitudinal strain [49] to construct a noninvasive pressure–strain loop. The patient‐specific LV pressure curve was constructed by adjusting the standard LV pressure curve to the duration of the isovolumic and ejection phases defined by the timing of valvular events (marked by previously recorded Doppler imaging). Strain and pressure data were synchronized using the ECG‐derived R‐wave onset, providing pressure‐strain loop curves whose areas expressed the global work index, evaluated from mitral valve closure to mitral valve opening. Among the myocardial work components, constructive work (mmHg %) of myocardial segments was defined as the sum of the effective work produced during segmental shortening in systole and during the lengthening of isovolumic relaxation time, which is a representative of the total amount of energy actively contributing to systolic work, expressed by the formula: global constructive work = systolic shortening + lengthening during isovolumic relaxation time. In contrast, wasted work (mmHg %), computed as the sum of the energy spent for the lengthening in systole and shortening in isovolumic relaxation time, corresponds to the energy loss spent during ineffective work, expressed by the formula: global wasted work = systolic lengthening + shortening during isovolumic relaxation time. The ratio of constructive work to the sum of constructive and wasted work represents myocardial work efficiency (%): global work efficiency = constructive myocardial work/(constructive myocardial work + wasted myocardial work). Global values were obtained as averages of all segmental values of the myocardial work parameters, including the global work index, global constructive work, global wasted work and global work efficiency.

### Statistical analysis

All statistical analyses were performed using the Statistical Package for Social Science (SPSS) software version 28 (IBM Inc.). The distribution of variables was assessed using the Kolmogorov–Smirnov test, and all variables showed a normal distribution. Data are expressed as means ± standard deviation for continuous variables and as absolute (percentage) values for discrete variables. Contingency table chi‐square tests and analysis of variance (with Bonferroni correction for multiple comparisons) were used to test for between‐group differences in nonparametric and parametric variables. Statistical significance was set at *p* value < 0.05.

## Results

In the lapse of time considered and according to our inclusion and exclusion criteria, we enrolled 69 patients with PDB [mean number of skeletal sites involved by PDB: 2.2 ± 1.6; monostotic/polyostotic PDB: 31 (44.9%)/38 (55.1%); tALP serum levels: 102.3% ± 69.2%] and in 115 controls. All patients with PDB enrolled for this study had been previously treated with at least one zoledronic acid infusion (mean number of previous treatments: 3.1 ± 1.4; average time from the last treatment: 12.8 ± 6.6 months; minimum timeframe from the last treatment: 6 months). The anthropometric and clinical characteristics of the study population are presented in Table 1. According to the enrolment criteria, no significant difference was reported in the PDB and controls for sex, age, BMI, BSA and heart rate. History of arterial hypertension was reported in 46 (66.7%) patients with PDB and 71 (61.7%) controls; however, the difference was not statistically significant (*p* = 0.32). The treatment of arterial hypertension among patients with PDB and controls is shown in Table 1. None of the patients receiving beta‐blocker treatment were included in the study. No statistically significant differences were observed between the groups.

**Table 1:** Anthropometric and clinical characteristics of PDB patients and control subjects.

	Patients with PDB	Controls
*N*	69	115
Age (years)	67.5 ± 7.7	65.7 ± 6.3
Male (*n*; %)	40; 58.0	61; 53.0
Body mass index (kg/m^2^)	22.6 ± 3.2	21.8 ± 2.8
Body surface area (m^2^)	1.75 ± 0.34	1.70 ± 0.34
Systolic blood pressure (mmHg)	135.6 ± 15.2	134.8 ± 17.1
Diastolic blood pressure (mmHg)	80.5 ± 8.6	80.7 ± 10.0
Mean blood pressure (mmHg)	98.9 ± 9.5	98.7 ± 11.0
Heart rate (bpm)	69 ± 11	67 ± 10
History of arterial hypertension and on medical treatment (*n*; %)	46; 66.7	71; 61.7
On treatment with angiotensin converting enzyme inhibitors (*n*; %)	20; 43.5	31; 43.7
On treatment with calcium channel blockers (*n*; %)	12; 26.0	17; 23.9
On treatment with thiazide diuretics (*n*; %)	9; 19.5	14; 19.7
On treatment with angiotensin receptor blockers (*n*; %)	5; 10.9	9; 12.6

*Note*: Data are expressed as mean ± standard deviation or absolute number; percentage for continuous and categorical variables, respectively.

Abbreviations: bpm, beats per minute; mmHg, millimetres of mercury; PDB, Paget's disease of bone.

The standard and speckle‐tracking echocardiographic parameters measured in the study population are summarized in Table 2. Among the standard echocardiographic parameters, interventricular septal diameter, pulsed‐wave tissue Doppler, relative wall thickness, LV mass index, *E*/*e*′ ratio, right ventricular trasversal diameter and systemic vascular resistance were significantly higher in patients with PDB compared to those in controls, whereas the LVEF, stroke volume and cardiac output were significantly lower in patients with PDB compared to those in controls (*p* < 0.05 in all cases).

**Table 2:** Standard and speckle‐tracking echocardiographic parameters in PDB patients and control subjects.

Parameters	Patients with PDB	Controls
Number	69	115
End‐diastolic interventricular septum thickness (mm)	10.2 ± 1.7^**^	9.1 ± 1.4
End‐diastolic posterior wall thickness (mm)	8.7 ± 1.6^*^	8.1 ± 1.4
End‐diastolic left ventricle diameter (mm)	47.8 ± 5.9	48.3 ± 5.9
End‐diastolic volume (mL)	109.6 ± 31.1	111.1 ± 28.6
End‐systolic volume (mL)	38.4 ± 17.8	39.3 ± 17.1
Left ventricle mass index (g/m^2^)	96.0 ± 30.6^*^	84.9 ± 21.2
Relative wall thickness	0.38 ± 0.06^*^	0.35 ± 0.07
Peak velocity of early diastolic transmitral flow/peak velocity of late transmitral flow ratio	0.90 ± 0.30	0.87 ± 0.21
Peak velocity of early diastolic transmitral flow/peak velocity of early diastolic mitral annular motion as determined by pulsed wave Doppler ratio	9.2 ± 2.6^*^	8.3 ± 2.2
Left atrial volume index (mL/m^2^)	32.8 ± 12.7	33.2 ± 11.6
Right atrial volume index (cm/m^2^)	19.7 ± 6.7	19.4 ± 4.4
Tricuspid annular plane systolic excursion (mm)	22.9 ± 4.1	22.9 ± 3.2
Right ventricular trasversal diameter (mm)	36.1 ± 4.1^**^	34.3 ± 4.9
Tricuspid regurgitation (m/s)	2.2 ± 0.3	2.2 ± 0.2
Pulmonary arterial systolic pressure (mmHg)	27.5 ± 6.3	27.6 ± 5.4
Left ventricular ejection fraction (%)	60.7 ± 5.1^***^	62.8 ± 4.3
Stroke volume (mL)	64.9 ± 17.9^*^	74.0 ± 17.8
Cardiac output (L/min)	4.5 ± 1.5^*^	4.9 ± 1.5
Cardiac index (L/min/m^2^)	2.7 ± 1.1	2.9 ± 0.9
Systemic vascular resistance (dynes/s/cm^5^)	1809 ± 514^*^	1668 ± 556
Global longitudinal strain (%)	21.2 ± 2.6^*^	22.3 ± 2.2
Global work index (mmHg %)	2450 ± 489	2351 ± 433
Global constructive work (mmHg %)	2625 ± 516	2628 ± 435
Global wasted work (mmHg %)	136 ± 67	103 ± 78
Global work efficiency (%)	94.1 ± 2.1^*^	95.4 ± 2.9
Aortic valve calcification or sclerosis	26; 37.7^***^	23; 20.0
Mitral valve calcification or sclerosis	16; 23.2^*^	7; 6.1

*Note*: Data are expressed as mean ± standard deviation or absolute number; percentage for continuous and categorical variables, respectively.

Abbreviations: PDB, Paget's disease of bone.

* Statistically significant compared to control subjects (*p* value < 0.05).

** Statistically significant compared to control subjects (*p* value < 0.01).

*** Statistically significant compared to control subjects (*p* value < 0.001).

Using speckle‐tracking echocardiography, patients with PDB showed lower values of global longitudinal strain and global work efficiency than controls (*p* < 0.05).

Patients with PDB showed a higher prevalence of sclerosis and/or calcification of the aortic and mitral valves than controls (*p* < 0.05).

The analysis was repeated after excluding all participants who were currently using cardiac drugs (Table 3). Among the standard echocardiographic parameters, end‐diastolic interventricular septum thickness, end‐diastolic posterior wall thickness, peak velocity of early diastolic transmitral flow/peak velocity of early diastolic mitral annular motion as determined by the pulsed‐wave Doppler ratio, *E*/*e*′ ratio and systemic vascular resistance were significantly higher, whereas the LVEF, stroke volume, cardiac index and cardiac output were significantly lower in patients with PDB than those in controls. Among the speckle‐tracking parameters, global longitudinal strain and global work efficiency were significantly lower, whereas global wasted work was significantly higher in patients with PDB than those in controls.

**Table 3:** Standard and speckle‐tracking echocardiographic parameters in all subjects, after excluding all those currently using cardiac drugs, classified intoPDB and control subjects.

Parameters	Patients with PDB	Controls
Number	39	69
End‐diastolic interventricular septum thickness (mm)	10.1 ± 1.7^**^	8.8 ± 1.1
End‐diastolic posterior wall thickness (mm)	8.7 ± 1.4^*^	8.1 ± 1.4
End‐diastolic left ventricle diameter (mm)	47.8 ± 6.2	49.6 ± 5.6
End‐diastolic volume (mL)	104.8 ± 32.1	116.5 ± 28.4
End‐systolic volume (mL)	38.8 ± 18.0	40.4 ± 14.9
Left ventricle mass index (g/m^2^)	93.1 ± 34.2	87.1 ± 20.4
Relative wall thickness	0.38 ± 0.06^*^	0.33 ± 0.07
Peak velocity of early diastolic transmitral flow/peak velocity of late transmitral flow ratio	0.87 ± 0.22	0.93 ± 0.24
Peak velocity of early diastolic transmitral flow/peak velocity of early diastolic mitral annular motion as determined by pulsed wave Doppler ratio	8.9 ± 1.9^*^	7.9 ± 2.1
Left atrial volume index (mL/m^2^)	31.5 ± 14.0	28.6 ± 9.0
Right atrial volume index (cm/m^2^)	17.6 ± 5.1	18.2 ± 6.4
Tricuspid annular plane systolic excursion (mm)	23.3 ± 3.6	23.2 ± 3.0
Right ventricular trasversal diameter (mm)	35.9 ± 4.2	34.3 ± 5.7
Tricuspid regurgitation (m/s)	2.2 ± 0.3	2.2 ± 0.2
Pulmonary arterial systolic pressure (mmHg)	27.6 ± 6.7^*^	27.4 ± 5.1
Left ventricular ejection fraction (%)	60.5 ± 5.8^*^	63.1 ± 4.0
Stroke volume (mL)	60.3 ± 17.3^**^	80.5 ± 17.9
Cardiac output (L/min)	4.2 ± 1.4^**^	5.3 ± 1.7
Cardiac index (L/min/m^2^)	2.6 ± 0.7^*^	3.1 ± 0.9
Systemic vascular resistance (dynes/s/cm^5^)	1945 ± 579^*^	1410 ± 410
Global longitudinal strain (%)	20.8 ± 2.3	22.4 ± 2.1
Global work index (mmHg %)	2469 ± 507	2312 ± 398
Global constructive work (mmHg %)	2636 ± 551	2605 ± 404
Global wasted work (mmHg %)	147 ± 87^**^	83 ± 5
Global work efficiency (%)	94.0 ± 2.1^*^	96.1 ± 2.2
Aortic valve calcification or sclerosis (*n*; %)	16; 42.1^**^	4; 14.8
Mitral valve calcification or sclerosis (*n*; %)	11; 28.9^**^	1; 3.7

*Note*: Data are expressed as mean ± standard deviation or absolute number; percentage for continuous and categorical variables, respectively.

Abbreviations: PDB, Paget's disease of bone.

* Statistically significant compared to control subjects (*p* value < 0.05).

** Statistically significant compared to control subjects (*p* value < 0.01).

***Statistically significant compared to control subjects (*p* value < 0.001).

According to scintigraphic data, 31 patients with PDB (male:female 14:17, mean age 66.7 ± 8.4 years, BMI 23.0 ± 3.36 kg/m^2^, BSA 1.76 ± 0.31 m^2^, systolic BP [SBP] 132.6 ± 17.2 mmHg, diastolic BP [DBP] 80.3 ± 10.0 mmHg, mean BP [MBP] 97.7 ± 11.0 mmHg, heart rate 72.0 ± 11.4 bpm) had a monostotic disease and 38 patients with PDB (male:female 26:12; mean age 68.2 ± 7.2 years, BMI 22.3 ± 3.1 kg/m^2^, BSA 1.74 ± 0.40 m^2^, SBP 137.9 ± 13.3 mmHg, DBP 80.6 ± 7.5 mmHg, MBP 99.7 ± 9.5 mmHg, heart rate 67.3 ± 10.3 bpm) had a polyostotic disease. No significant differences were observed in the standard and speckle‐tracking echocardiographic parameters between patients with PDB classified according to monostotic and polyostotic disease.

According to serum tALP levels, 21 patients with PDB (male:female 11:10, mean age 67.4 ± 7.1 years, BMI 21.8 ± 3.2 kg/m^2^, BSA 1.63 ± 0.28 m^2^, SBP 131.2 ± 10.7 mmHg, DBP 76.1 ± 5.1 mmHg, MBP 94.5 ± 5.4 mmHg, heart rate 74.6 ± 11.3 bpm) showed elevated serum tALP levels, whereas the remaining 48 patients with PDB showed tALP serum levels within the normal range (male:female 30:18, mean age 67.5 ± 7.5 years, BMI 22.9 ± 3.3 kg/m^2^, BSA 1.80 ± 0.35 m^2^, SBP 135.5 ± 14.7 mmHg, DBP 82.4 ± 9.1 mmHg, MBP 100.8 ± 9.1 mmHg, heart rate 67.1 ± 10.8 bpm). No significant differences were observed in the standard and speckle‐tracking echocardiographic parameters between patients with PDB and elevated serum tALP levels and those with serum tALP levels within the normal range.

Eight patients with PDB (male:female 5:3, mean age 68.4 ± 3.6 years, BMI 24.6 ± 1.2 kg/m^2^, BSA 1.92 ± 0.23 m^2^, SBP 136.7 ± 18.6 mmHg, DBP 80.0 ± 8.9 mmHg, MBP 98.9 ± 10.5 mmHg, heart rate 63.0 ± 11.8 bpm) presented mutations in *SQSTM1*. We observed three patients with PDB with *P392L^SQSTM1^* mutation, one patient with PDB with *M401V^SQSTM1^* mutation, one patient with PDB with G425E*^SQSTM1^* mutation, one patient with PDB with Y383X*^SQSTM1^* mutation and two patients with PDB with A427D *^SQSTM1^* mutation. All *SQSTM1* mutations were within exons 7 and 8 of the gene [50]. The remaining 61 patients with PDB (male:female 35:26, mean age 67.4 ± 8.6 years, BMI 22.3 ± 4.2 kg/m^2^, BSA 1.72 ± 0.38 m^2^, SBP 135.5 ± 14.6 mmHg, DBP 80.6 ± 8.8 mmHg, MBP 98.9 ± 9.5 mmHg, heart rate 70.2 ± 11.8 bpm) showed wild‐type *SQSTM1*. No significant differences were observed in the standard and STE parameters measured in patients with PDB and the *SQSTM1* mutation and patients with PDB and wild‐type *SQSTM1*.

## Discussion

In recent years, clinical and experimental studies have described the simultaneous and growing incidence of both cardiovascular and metabolic bone disorders, raising questions about whether each disorder can influence the risk of the others [51]. The results of this case–control study showed that cardiac impairment was more prevalent in patients with treated PDB than in those without this disease, matched for sex, age, BMI and history of hypertension/BP. This demonstrates that patients with PDB without clinical evidence and personal history of CVDs show early and subclinical cardiac impairment, characterized by systo‐diastolic dysfunction, with higher LV‐filling pressures [52], lower ejection fraction, concentric LV remodelling and a higher prevalence of cardiac valve sclerosis and calcifications compared to controls with identical clinical characteristics and matched for age, sex, BMI and history of arterial hypertension. The results of the standard echocardiographic examination are also supported by the advanced speckle‐tracking echocardiographic technique, confirming worse LV systolic function by the lower global longitudinal strain and global work efficiency values. These results remained significant even after excluding all patients who were currently using cardiac drugs. Interestingly, the metabolic activity of PDB, its extension or the occurrence of *SQSTM1* mutations in patients with PDB do not significantly influence echocardiographic findings, which appear to be linked only to the disease itself.

These results seem to be in line with LV systo‐diastolic dysfunction, most likely due to the increased cardiac afterload, in turn leading to the significantly higher systemic vascular resistance in patients with PDB. In this phase, the LV responds to chronic elevations in afterload by concentric hypertrophy, causing increased wall thickness (i.e., LV mass), which maintains or slightly affects cardiac output (i.e., LVEF, cardiac index and stroke volume) through a regulatory mechanism [53]. The results of this study prove that the LV systo‐diastolic dysfunction and acquired valvulopathies should be considered relevant comorbidities in patients with PDB and pave the way for new and intriguing perspectives for clinical and experimental research, suggesting the occurrence of a specific pagetic heart disease (PHD).

From a clinical perspective, the impact of PHD on the quality of life and lifespan of patients with PDB needs to be quantified through ad hoc studies on holistic PDB patient care. Indeed, LV remodelling is a major and independent risk factor for cardiovascular morbidity, mortality and all‐cause mortality, including neurological pathologies [54]. Moreover, the impact of PDB treatments, including amino bisphosphonates and cholecalciferol, on the heart function should be globally analysed [55, 56]. Even if cholecalciferol does not have a specific role in the management of PDB, biological follow‐up focuses on its serum levels, especially in the context of treatment with bisphosphonates, as recommended in Italy for PDB management according to the position paper for PDB published by the Italian Society of Osteoporosis, Mineral Metabolism and Skeleton Diseases (Società Italiana Osteoporosi e Malattie del Metabolismo Minerale e Dello Scheletro – SIOMMMS) [39] and the Italian Drug Agency (Agenzia Italiana del Farmaco – AIFA) [57].

From an experimental perspective, identifying the etiopathogenic mechanisms underlying PHD is an intriguing challenge.

Indeed, heart‐bone‐vessel axis dysregulation linked to pathognomonic alterations in the bone turnover of PDB [7] can justify the echocardiographic findings. Bone‐regulating hormones, nutrients and turnover markers influence several aspects of cardiometabolic health, including body composition, cardiovascular function and glycaemic control [14]. First, dysregulation of the RANKL/OPG system, which causes vascular calcification, leads to a reduction in vascular wall elasticity, greater arterial stiffness and increased systemic vascular resistance [58]. Furthermore, paediatric osteoclasts exhibit high levels of oxidative stress [59], which, in turn, represents a strong atherogenic mechanism that increases arterial stiffness [60]. Additionally, the influence of bone metabolism on energy balance may be relevant to CVDs [61]. An intriguing hypothesis is that hormones regulating skeletal turnover (i.e., 25OH vitamin D [25OHD] and parathyroid hormone) and/or those secreted by bone cells under physiological and pathological conditions (i.e., fibroblast growth factor 23) could play a significant role in the pathogenesis of cardiovascular disorders [16, 62, 63]. Finally, there is a major concern regarding the role of Wnt signalling and sclerostin in the pathogenesis of CVDs and heart valve calcification [64].

To the best of our knowledge, we cannot exclude the role of the bone‐heart axis in the pathogenesis of PHD.

Our study has several strengths and limitations. Due to its observational nature, it cannot elucidate the causal relationship between PDB and echocardiographic findings but can only demonstrate a connection. Moreover, the absence of a baseline examination for all patients before commencing bisphosphonate treatment conceals the pathogenesis of myocardial function found in this study and does not explain the positive or negative outcomes of the treatment. In addition, the strict inclusion criteria ruled out all patients with PDB affected by CVD, limiting the number of subjects with *SQSTM1* mutations available for comparison. The poor sample size may have masked some significant differences between patients with PDB and without *SQSTM1* mutations, for which a wider study must be properly designed. In contrast, to the best of our knowledge, this is the first study to compare and match two populations according to major clinical features and confounders such as CVD, age, BMI and personal history of arterial hypertension. In addition, the mechanisms of abnormal bone remodelling in PDB can provide new insights into the pathophysiology and comorbidities of other bone disorders [63, 64]. As such, our study paves the way for further investigations of cardiac impairment and the impact of amino‐bisphosphonate treatment in cases of skeletal disorders [65, 66, 67, 68, 69].

In conclusion, the results of our study demonstrate that LV systo‐diastolic dysfunction and acquired valvulopathies should be considered relevant comorbidities in patients with PDB, suggesting the occurrence of a specific cardiopathy, PHD. Although occasional cardiac complications have been described [2, 70], the lack of broad echocardiographic data precludes the opportunity to suggest a tailored evaluation. Based on these findings, careful evaluation using standard and advanced echocardiographic examinations must be performed during all stages of PDB patient assessment.

Given the lack of new information of CVD in a recently published national position paper from 2024 [39], this study provides a stimulus for further work in these areas. New prospective and larger studies are warranted to confirm our data, quantify the clinical impact of PHD on the prognosis of patients with PBD and evaluate the need for cardiologic management in PHD.

## Author Contributions

**Alfonso Giaquinto**: Conceptualization; investigation; writing—original draft. **Veronica Abate**: Writing—original draft. **Anita Vergatti**: Data curation; visualization. **Riccardo Muscariello**: Investigation. **Adelaide Iervolino**: Investigation. **Martina Pucci**: Investigation. **Guido Cavati**: Investigation; data curation. **Filippo Pirrotta**: Data curation; software. **Gianpaolo De Filippo**: Writing—review and editing; validation. **Roberta Esposito**: Writing—review and editing; supervision. **Lanfranco D'Elia**: Formal analysis. **Daniela Merlotti**: Investigation; formal analysis. **Luigi Gennari**: Project administration; resources. **Domenico Rendina**: Resources; funding acquisition; methodology.

## Conflict of interest statement

The authors declare no conflicts of interest. All co‐authors have read and agreed to the contents of the manuscript and have no financial interests.

## Funding information

The research leading to these results has not received any funding.

## Ethics statement

All protocols conducted in this study were approved by the Federico II University Ethics Committee (approval number: 117/2015).

## Data Availability

Data supporting the findings of this study are available upon request from the corresponding authors.
